# The State of Lunacy in Scotland

**Published:** 1863-07

**Authors:** 


### Art. IX.—THE STATE OF LUNACY IN SCOTLAND.

The fifth Annual Report of the Commissioners of Lunacy for Scotland affords information of the state of lunacy in that portion of the kingdom in 1861, and of the progress of legislation for the insane and operations of the Commissioners, together with the changes among the registered lunatic population, in 1862.

In the latter year an Act was passed "To make further provisions respecting lunacy in Scotland," and in the following summary of its provisions we shall adhere almost verbatim to the abstract given in the Report. This Act (25th and 26th Vict. c. 54) modifies in several important particulars the original Lunacy Act (20th and 21st Vict. c. 71). It extends the definition of the term "lunatic," which it declares shall now embrace every person certified by two medical persons to be "a lunatic, an insane person, an idiot, or a person of unsound mind." "This important change," say the Commissioners, "makes the definition of lunacy in the Scotch law identical with that of the English statute, and will, we believe, tend to the removal of obstacles which hitherto have occasionally interfered with the prompt and proper treatment of the insane. At the same time we trust that the declaration required by the medical certificates, that the patient is 'a proper person to be detained under care and treatment,' will prevent undue recourse being had to seclusion in asylums."

The Amendment Act also provides for the recognition of lunatic wards in poorhouses under certain restrictions; and further, it empowers the Commissioners to grant special licences to occupiers of private houses for the reception and detention therein of lunatics, not exceeding four in number, without the exaction of any licence-fee. This power, in connexion with that also conferred on the Commissioners of sanctioning the discharge of patients from asylums on trial or probation, will, they hope, tend to facilitate the restoration to ordinary life of many patients for whom asylum treatment and discipline are not absolutely required. Other facilities in the disposal of patients are afforded by the authority which this Act confers on the Commissioners, of granting licences without fee to charitable institutions established for the care and training of imbecile children, and supported in whole or in part by private subscriptions ; and likewise by the power it bestows on them of sanctioning the transfer of patients from one asylum to another without the order of the sheriff.

A further important modification in the manner of disposing of pauper lunatics introduced by the Act, is the relaxation of the stringent enactment which required District Boards of Lunacy to provide accommodation for their pauper lunatics within their own districts, either in Public or District Asylums. For the future, agreements and arrangements may, with the sanction of the Commissioners, be made "for the reception and detention of all or any of the pauper lunatics of any district, county, or parish, in any public, private, district, or parochial asylum or hospital, within or beyond the limits of such district, county, or parish." But while the basis on which accommodation may be provided is thus extended, it is enacted that the Court of Session, in the event of a District Board failing to take such steps as the Home Secretary, on the representation of the Commissioners, may consider requisite for the accommodation of the pauper lunatics of the district, shall, on due cause shown, appoint a person at whose sight and instance the whole power and duties of such District Board, relative to the providing of accommodation, shall be performed.

Additional security for the proper care of pauper lunatics is provided by the authority given to the Commissioners to take whatever steps may be necessary for the removal of such patients to asylums, in case of the refusal or failure of Parochial Boards to carry out, in this respect, the requirements of the statute.

The procedure for placing patients in asylums has also in some degree been modified by the Act. The petitioner is now called on to declare the capacity in which he stands to the patient; and the convenience of the public has been consulted by extending the period during which a certificate of emergency is valid, from one to three days.

The disposal of dangerous lunatics has been facilitated and rendered less expensive by authorizing the sheriff to permit the inspector of poor, within a certain stage of proceedings, to take charge of the patient, provided such arrangements be made for his safe custody as are satisfactory to the sheriff.

"The spirit of liberal legislation," say the Commissioners, "which the foregoing details show to pervade this Act, further appears in a clause which permits persons to place themselves voluntarily under treatment in asylums. Unfortunately, however, the procedure followed is too complicated to allow us to hope that the provisions of this clause will be taken advantage of, except on very rare occasions. The remaining clauses refer chiefly to the disposal of criminal lunatics, and do not at present call for special notice."

The increase in the number of pauper lunatics in 1861, as in 1860, was remarkably small. In 1858 it amounted to 243; in 1859, to 246; in 1860, to 31; and in 1861, to 32.

The distribution of the insane in Scotland on the 1st January, 1862, was as follows:—

PUBLIC AND DISTRICT ASYLUMS.

| | |
|---|---|
| Number of patients | 2820 |
| Males | 1403 |
| Females | 1417 |
| Increase since the 1st January, 1861 | 108 |
| Supported by private funds | 800 |
| „ by parochial rates | 2020 |

On the 1st January, 1861, the numbers supported by private funds and parochial rates, were respectively 766 and 1946. It thus appears that during 1861 an increase of 34 had taken place in the number of private patients, and one of 74 in that of paupers.

PRIVATE ASYLUMS.

| | |
|---|---|
| Number of patients | 921 |
| Males | 397 |
| Females | 524 |
| Increase since the 1st January, 1861 | 14 |
| Supported by private funds | 231 |
| „ by parochial rates | 690 |

On the 1st January, 1861, the numbers supported by private funds and parochial rates were respectively 224 and 683. It thus appears that during 1861 an increase of 7 had taken place in the number of private patients, and one of 7 in that of paupers.

POOR-HOUSES.

| | |
|---|---|
| Number of patients | 838 |
| Males | 335 |
| Females | 503 |
| Decrease since the 1st January, 1861 | 5 |

The whole of these patients were chargeable to their parishes.*

SINGLE PATIENTS.

| | |
|---|---|
| *Pauper.*—Number of patients | 1741 |
| Males | 769 |
| Females | 972 |
| Decrease since the 1st January, 1861 | 46 |
| *Living with relatives :—* | |
| Males | 618 |
| Females | 720 |
| *Placed with strangers :—* | |
| Males | 139 |
| Females | 195 |
| *Living alone :—* | |
| Males | 12 |
| Females | 57 |

*Private.*—"During our first investigation into the condition of the insane not placed in establishments," say the Commissioners, "we acquired knowledge of the existence in private dwellings of 1887 insane persons—1041 males, and 846 females—supported from private funds. In later inspections, however, we have purposely omitted to make any particular inquiries regarding such patients, as being for the most part resident with their own families, they are not subject to statutory visitation. At the beginning of 1862, there were only 21 private single patients who, in conformity with the forty-first section of the Lunacy Act, were placed under the order of the sheriff, in the houses of strangers. From the great uncertainty

* A misprint occurred in our notice of the Fourth Annual Report of the Commissioners in Lunacy for Scotland, which we would here correct. *See* vol. ii., p. 682, second line from the bottom, where, in place of "With the exception of *the* females, the whole of these patients were maintained by parishes," read, "With the exception of *two* females," &c.

which under these circumstances must accompany any estimate of the number of unreported private patients, we now think it more advisable not to take them in account."

SUMMARY.

| | | |
|---|---|---|
| Total number of Insane under official cognizance on the 1st January, 1862 | 6341 | |
| Males | | 2912 |
| Females | | 3429 |
| *Supported by private funds:—* | | |
| Males | | 519 |
| Females | | 533 |
| | | 1052 |
| *Supported by parochial rates:—* | | |
| Males | | 2393 |
| Females | | 2896 |
| | | 5289 |
| Increase of private patients since 1st January, 1861 | | 39 |
| Increase of paupers ditto | | 32 |
| Total increase | | 71 |

"To what cause the comparatively slow growth of pauper lunacy is to be ascribed," say the Commissioners, "we are not prepared to offer an opinion; but we scarcely venture to hope that the result of future years will be found as favourable." On the 1st January, 1858, the number of *pauper* lunatics amounted to 2953, of *private* to 1012; at the same date in 1862 the number of *pauper* lunatics was 3548, of *private* 1031. The nearly stationary amount of private lunacy is ascribed "principally to the impoverishing operation of mental disease, through which a considerable number of private patients are each year converted into paupers; and the greater growth of pauper lunacy partly to the cause alluded to, and partly to a diminished mortality from better care and treatment."

The prevalence of insanity would appear to be greater in Scotland than in England, when a comparison is instituted between the official returns of the English and Scotch Lunacy Boards. But the English returns do not take account of single patients, and the English Commissioners do not possess so accurate a knowledge as the Scotch Commissioners of the amount of pauper lunacy within the limits of their jurisdiction. Every parish in Scotland is visited on an average once a year, either by the Medical or Deputy Commissioners; consequently, there is good reason to believe that the number of unreported pauper lunatics is much less in Scotland than in England. The Commissioners are therefore of opinion that "any seeming excess of lunacy in Scotland is not so much due to a larger proportion of insane in the population, as to more copious and accurate returns."

The Commissioners discuss the policy of relatives being permitted to remove unrecovered pauper lunatics from asylums by simply withdrawing their names from the poor-roll. "It might at first sight appear harsh and despotic," they say, "to place impediments in the way of the poor removing their insane relatives from asylums, for the purpose of maintaining them at home, which are not equally applicable to the non-pauper classes. But the main point for consideration here is, whether the relatives of lunatics, by placing them under treatment at the public expense, do not come under a moral obligation to leave them in the asylum so long as well-founded hopes are entertained of their recovery, or of their receiving special benefit from the means of treatment therein employed. A like argument, it will be observed, is not applicable to private patients who have never taken advantage of public relief. The question is not without its difficulties; but, on the whole, our experience would lead us to recommend that the removal of unrecovered pauper lunatics should be made dependent on the concurrence of the medical superintendent of the asylum; or, alternatively, on that of this Board."

An excess of female pauper lunacy in Scotland, it is pointed out, is probably not so much due to a greater disposition of the female sex to insanity, as to the larger source from which the supply of female pauper patients is derived. Among registered paupers the proportion of males to females is as 100 to 297. Among private patients the proportion of male to female lunatics placed in asylums is as 100 to 106; the proportion of males to females in the general population being 100 to 111.

The Commissioners testify to continued improvement in the condition both of public and private asylums. A question arose, in one instance, of patients not being allowed to hold any communication with the Board, or the outer world, except with the cognizance of the asylum authorities. The injudicious and injurious character of such a regulation is justly indicated by the Commissioners. A probable evil is pointed out as likely to arise from the wording of the Amendment Act in respect to the detention of patients in poor-houses. The Act authorizes the

Commissioners to license lunatic wards in poor-houses for the reception and detention of "such pauper lunatics only who are not dangerous, and do not require curative treatment." This would tend to encourage the belief that safe detention is all that is required for the proper care and treatment of this class of the insane. The Commissioners rightly state that "under judicious management, and provided with proper means of occupation, the great mass of the insane are capable of being actively and usefully employed, and in a manner calculated to afford them positive enjoyment. In these respects there is no difference between the so-called dangerous and non-dangerous classes, or between the curable and incurable. It follows, therefore, that before we can separate patients into distinct categories, for which different kinds of accommodation should be provided, we must regard the incurable as beyond the pale of humane and enlightened treatment. Such a result would be most deplorable, but it is one which is directly encouraged by the system of attaching lunatic wards to poor-houses."

The opposite table shows the admissions into public and private asylums and the lunatic wards of poor-houses, the average number of patients resident, the discharges and deaths in 1861 and 1862, with the average of the five years 1858–1862.

But one school for idiotic and imbecile children, that of Baldovan, is at present in operation in Scotland. A new school is, however, in process of erection, by the Society for the Education of Imbecile Children, in the vicinity of the Larbert Station of the Scottish Central Railway. This establishment is destined for the accommodation of about 50 cases, including apartments for the better class of children. The cost of the building now being erected will be about £4000; but the plans provided for an extension so as to accommodate 200 cases, at a total cost of £10,000.

The Board of Lunacy was, in the course of 1862, deprived by death of the services of the chairman, Mr. Forbes Mackenzie of Portmore. "In every question of difficulty," say the Commissioners, "we were greatly indebted to his sound judgment and practical good sense; and on all occasions we derived much assistance from his accurate business habits." The office of Deputy-Commissioner, vacant by the death of Dr. Cockburn, has been, "after considerable delay," bestowed on Dr. George Paterson.

| 1861. ASYLUMS. | Admitted. | | Average Number Resident. | | Discharged. | | | | Deaths. | |
|---|---|---|---|---|---|---|---|---|---|---|
| | | | | | Recovered. | | Not Recovered. | | | |
| | M. | F. | M. | F. | M. | F. | M. | F. | M. | F. |
| PUBLIC.—1861 . . . | 489 | 532 | 1374·0 | 1388·0 | 195 | 219 | 121 | 152 | 120 | 103 |
| 1862 . . . | 435 | 464 | 1405·0 | 1418·0 | 152 | 197 | 124 | 147 | 147 | 107 |
| *Average of Five Years,* 1858-62 . . . . | 445·6 | 493·4 | 1338·1 | 1307·0 | 162·8 | 204·2 | 128·8 | 135·6 | 124·5 | 99·2 |
| PRIVATE.—1861 . . . | 112 | 176 | 388·5 | 524·0 | 30 | 66 | 29 | 76 | 28 | 39 |
| 1862 . . . | 117 | 156 | 405·0 | 518·0 | 27 | 56 | 37 | 53 | 42 | 45 |
| *Average of Five Years,* 1858-62 . . . . | 119·0 | 179·8 | 372·6 | 501·9 | 38·4 | 68·0 | 30·0 | 49·0 | 31·6 | 38·2 |
| POORHOUSES.—1861 . | 152 | 189 | 339·0 | 495·0 | 65 | 94 | 31 | 40 | 54 | 45 |
| 1862 . | 174 | 192 | 344·5 | 505·5 | 71 | 73 | 64 | 60 | 36 | 59 |
| *Average of Five Years,* 1858-62 . . . . | 160·8 | 195·6 | 331·4 | 486·2 | 62·2 | 90·4 | 37·6 | 44·2 | 51·6 | 48·4 |

